# Limb Salvage vs. Amputation: Factors Influencing the Decision-Making Process and Outcomes for Mangled Extremity Injuries

**DOI:** 10.7759/cureus.30817

**Published:** 2022-10-28

**Authors:** Mohammad Waseem Beeharry, Thomas Walden-Smith, Komal Moqeem

**Affiliations:** 1 Trauma and Orthopaedics, Barts Health NHS Trust, London, GBR; 2 Orthopaedics and Trauma, King's College Hospital NHS Foundation Trust, London, GBR; 3 Internal Medicine, Royal Surrey County Hospital, Guildford, GBR

**Keywords:** mangled limb, amputation, complex limb injuries, poly trauma, limb-salvage

## Abstract

In the setting of acute severe limb injury, the clinical decision to either attempt limb salvage or to perform a primary amputation presents a significant challenge to the trauma team. The initial step in the management of a mangled limb is invariably resuscitation and stabilisation of the patient and an evaluation of the limb. However, the decision-making process on whether to amputate vs attempt limb salvage is dependent on a range of complex factors. This includes assessing the degree of injury to the components of the limb architecture, essential skeletal stability, soft tissues, vasculature, and neurological structures. Whether or not the patient would survive an attempt to limb salvage is of course not the only variable to be taken into account. The likely and expected outcomes of attempted salvage in each individual case must be considered and furthermore, what the acceptable side-effect profile including the risk of failure would be for each individual patient should be assessed against the importance, real or perceived, that limb function is maintained. Finally, the patient's choice should also be taken into account alongside their occupation and pre-morbid functional status. How the surgeon makes this life-changing, or life-threatening decision, is of great clinical significance, and there are myriad scoring systems published that purport to assist in this matter. However, the changing structures of the trauma system, expansion and advancement of skillsets and technology means an updated review is required to help weigh up the challenging decision of limb amputation vs salvage, which usually takes place in a time-pressured and highly emotional emergency setting. An evidence-based, standardised structure to assist in these calculations could support surgeons and improve outcomes for these patients.

## Introduction and background

In the setting of acute severe limb injury, the clinical decision to either attempt limb salvage or to perform a primary amputation presents a significant challenge to the trauma team. There is currently no consensus within the literature on whether one management route is superior to the other, although there is an agreement that the decision-making process is influenced by a multitude of variables [1]. These include the assessment of the grade of injury, patients’ premorbid and current physiological status, availability of resources, the skillset of the trauma team, and above all the patient’s priorities in terms of both their short and long-term outcomes [2]. There is no simple algorithm which can be employed that weighs in all these variables and definitive management remains a predicament as complex limb injuries often occur as a result of polytrauma with each case presenting with its unique set of challenges and patient expectations. 

The decision-making dilemma has been further complicated in recent years. Whilst traditional scoring systems can aid in the assessment of limb injuries, the implementation of trauma networks across England and Wales is rapidly changing the potential salvageability of a complex limb injury. A major trauma team now has more advanced resources and skills available leading to a greater number of limb salvage attempts in patients that would historically have undergone an amputation. 

Revised and updated evidence-based clinical decision tools are currently not available but despite this, it is of paramount importance to correctly assess and identify the subset of patients who would benefit from a primary amputation. A failed attempt at salvage would result in secondary amputation and will subject the patient to increased morbidity in terms of physical, psychological, financial, and social burden [3]. Therefore, careful evaluation through a multidisciplinary approach is essential, with input from different specialities including anaesthetists, orthopaedics, plastics, and vascular surgery. This would aid the team in systematically analysing the relevant variables and arriving at a balanced decision on the feasibility of the operation and likelihood of successful limb salvage, with mindful consideration of the long-term functional and psychological expectations of the patient. 

## Review

Factors affecting assessment

A thorough evaluation of the severely injured limb is crucial as it may impact the eventual outcome and guide definitive care. The initial approach should be resuscitation of the patient through a primary survey according to the Advanced Trauma Life Support (ATLS) protocols in a sequential fashion and address any life-threatening injuries. Assessment of the severely injured limb is based on the degree of injury to the components of the limb architecture, soft tissues, vasculature and neurological structures. Severe injuries sustained to a combination of these components (at least three out of four components) that brings about any subsequent concerns for limb viability are referred to as a ‘mangled extremity’ [1].

Complex limb injuries often arise in the context of poly-trauma. Poly-traumatised patients are classified in relation to their physiological response and extent of injuries on presentation. Pape et al. have described these patients as either stable, borderline, unstable or in extremis based on their clinical status [4]. Limb salvage must only be attempted in those patients who are haemodynamically stable to tolerate the required surgical procedure and blood loss associated with the procedure. This poses a dilemma in the physiologically unstable patient (unstable or in extremis) as the physiologic burden and systemic effects of multiple surgeries required to execute limb salvage must be evaluated before determining whether or not to amputate. In patients with persistent haemodynamic compromise, the priority remains to save life rather than limb salvage [3]. As Scalea et al. note, an ischaemic limb does not constitute an instantaneous threat to life in contrast to an exsanguinating limb, which should be addressed promptly [5]. 

The concept of damage control orthopaedics (DCO), based on a therapeutic approach that prioritises physiological restitution over anatomic fixation, have been widely implemented to manage the critically injured patient with life threatening injuries [6]. In the acute phase, damage control is often achieved through temporizing measures such as by early rapid fracture stabilisation through external fixation, fasciotomies or temporary vascular shunting to prevent the lethal triad of hypothermia, coagulopathy and metabolic changes [7]. 

There are a number of indicators, particularly risk factors for adverse outcomes, which are associated with an inclination for amputation of the mangled extremity [5]. These include systemic factors such as age being over 50 years and persistent hypotension of less than 90 mmHg. Further indicators are related to the nature of the injury such as substantial and destructive soft tissue injury and wound contamination. Additionally, bony skeletal factors contribute to adverse outcomes, particularly with regard to Gustilo Anderson Type III comminuted fractures with a high likelihood for the need of bone grafting. 

Vascular factors, particularly prolonged tissue ischaemic time, are an important indicator of limb viability. The release of oxygen free radicals and the metabolic disturbances associated with ischaemia can have serious repercussions in terms of systemic reperfusion injuries after surgery [8]. Several studies have reported irreversible changes associated with a prolonged ischaemic time of greater than six hours. To allow swift restoration of limb circulation in the management of arterial vascular injuries, temporary shunts such as femoral-to-radial, radial-to-radial, and cross-limb shunts have been used to provide a connection between severed vascular ends [9]. Data from the National Trauma Database (NTDB) estimates amputation rates following upper and lower limb arterial injuries at 1.3% and 7.8%, respectively [10]. 

Factors affecting decision-making

Several predictive scoring systems, primarily identifying risk factors based on the severity of the aforementioned parameters, have been designed to support the surgeon in deciding between limb salvage versus amputation. These predicting systems, mostly addressing lower extremity injuries, include the Predictive Salvage Index (PSI), Mangled Extremity Syndrome Index (MESI), Mangled Extremity Severity Score (MESS), Limb Salvage Index (LSI), Hannover Fracture Scale (HFS), and Ganga Hospital Open Injury Severity Score (GHOISS). The MESS was further evaluated and modified by McNamara et al. to devise the nerve injury, ischemia, soft tissue injury, skeletal injury, shock, and age of patient (NISSSA) score [11]. Additionally, the NISSSA scoring system also incorporates neurological findings, particularly plantar sensation, as an arbitrating factor. A comparison of the different scoring systems is shown in Table 1.

**Table 1:** Comparison of the components of the different scoring systems MESI: Mangled Extremity Syndrome Index; MESS: Mangled Extremity Severity Score; PSI: Predictive Salvage Index; LSI: Limb Salvage Index; GHOISS: Ganga Hospital Open Injury Severity Score; NISSSA: Nerve injury, Ischemia, Soft-tissue contamination, Skeletal damage, Shock, and Age. Source: Akgun Demir and Karsidag, 2020 [12]

	MESI	MESS	PSI	LSI	NISSSA	GHOISS
Age	+	+			+	+
Shock	+	+			+	+
Warm Ischaemia Time	+	+	+	+	+	+
Bone Injury	+		+	+		+
Muscle Injury			+	+		+
Skin Injury	+			+		+
Nerve Injury	+			+	+	
Deep Vein Injury				+		
Skeletal/Soft Tissue		+			+	
Contamination					+	+
Time to treatment	+			+		
Co-morbidity	+					+

An exclusive scoring system is ineffective for both upper and lower extremities due to their anatomy. The smaller bulk of muscle and richer vascular supply of the upper limb is not comparable, as ischaemia time would be relatively more [13]. McNamara and colleagues retrospectively studied 24 patients sustaining open fractures of the tibia with Gustilo Anderson type IIIB and type IIIC and performed a comparison between the MESS and NISSSA scores. Although both the scoring systems were accurate and statistically significant (p<0.005) in predicting amputation, they found that the NISSSA score was more sensitive (81.8% versus 63.6%) and more specific (92.3 versus 69.2%) than the MESS [11]. 

Whilst they assist the surgeon in the decision-making process, these scoring systems are by no means infallible and cannot be used as a sole criterion when making decisions. They fail to take into account factors related to the patient's quality of life, pain, occupation, wishes, and socio-economic status [14]. To assess the clinical utility of these predictive scoring systems, 556 patients with high-energy lower-extremity injuries were prospectively studied by Bosse et al. as part of a multi-centre study called the Lower Extremity Assessment Project (LEAP). Their analysis showed that the five scoring systems were highly specific in predicting limb salvage potential when their scores were low. However, they failed to predict when amputation was warranted due to the low sensitivity of the indices [15]. The sensitivity and specificity of the five scoring systems of the LEAP study are shown in Table 2 [15].

**Table 2:** Sensitivity and specificity of the predictive scoring systems adapted from the LEAP study MESS: Mangled Extremity Severity Score; PSI: Predictive Salvage Index; LSI: Limb Salvage Index; HFS: Hannover Fracture Scale; NISSSA: Nerve injury, Ischemia, Soft-tissue contamination, Skeletal damage, Shock, and Age; LEAP: Lower Extremity Assessment Project

	MESS	PSI	LSI	NISSSA	HFS
Sensitivity	0.45	0.47	0.51	0.33	0.37
Specificity	0.93	0.84	0.97	0.98	0.98

A systematic review comparing the current scoring systems by Schiro et al. concluded that none of the mostly used scores (MESS, LSI, PSI, and NISSSA) in the literature showed reliability in discriminating the limb injuries requiring primary or secondary salvage and failed to predict the functional outcome following successful limb salvage. All of these scoring systems were developed prior to the implementation and reorganisation of the trauma network systems across England and Wales. With modern developments in trauma resuscitation, availability of resources and skills, and technological advances, these scoring systems need revision and updating to be more applicable [16]. 

Factors affecting outcomes

In a trauma setting, severe and complex injuries to the limbs require systematic and holistic evaluation of the overall disability of the patient. Surgical outcomes have been classically tracked by monitoring factors such as the development of osteoarthritis, radiological findings of fracture healing, and skeletal alignment as well as the presence of post-operative infection [17]. However, those traditional metrics could potentially compromise more important patient-related outcomes (PRO) such as psychological effect, socioeconomic status, quality of life, and a successful return to work [18]. Hence, the decision-making process, when considering a management plan should prioritise achieving a satisfactory outcome for the patient as a whole as opposed to focusing on treating the injuries in isolation. 

When considering an outcome-driven approach in deciding if the limb is to be salvaged or amputated in the lower extremity, recognition should be given to the significant difference between injuries above and below the knee. From a surgical perspective, salvage attempts for above-knee injuries are less complex than lower-knee injuries, owing to the thicker soft tissue envelope that often makes reconstruction easier. However, Dirschl and Dahners suggest that in terms of prosthetic function, a trans-tibial amputation is generally more favourable in contrast to a trans-femoral one [19]. Alongside the location of the injury, the need to undergo a secondary amputation is of significance as it influences both the functional and psychological aspects of the patient. A study by Williams et al. investigated the outcomes of patients undergoing early amputation (<48 hours) versus late amputations (>48 hours). They found that the latter had significantly more ipsilateral leg complications, longer lengths of hospital stay, and higher rates of local limb complications [20]. 

Data published in 2002 from another subset of the LEAP study measured the functional outcomes, using the Sickness Impact Profile (SIP) in terms of a self-reported health status assessment of function. Bosse et al. found no significant difference between the amputation and reconstruction groups at 24 months, albeit a subgroup of patients who had limb salvage was observed to have significantly more hospital re-admissions related to complications and a longer rehabilitation time to full weight-bearing status contributing to the morbidity of the patient [21]. A meta-analysis carried out by Busse and colleagues, comparing nine observational studies on early amputation versus complex limb salvage, concurred with the LEAP study with regards to additional surgeries, longer rehabilitation period and subsequent re-hospitalisation for the limb salvage patient [22]. They also found no significant differences in functional outcomes at least up to seven years post injury between the two groups. 

To assess the physical and psychological outcomes, Akula et al. performed a meta-analysis of 11 observational studies of post-traumatic amputees (769 patients) and limb salvage (369 patients), respectively, that used validated scoring systems such as Short Form (SF-36) and SIP to assess the quality-of-life. Their results indicated that salvaging the limb with reconstruction was much more appealing to patients and enhanced their quality of life psychologically, though the physical outcome was more or less the same [23]. In comparison, these outcomes differed in the military service population. The Military Extremity Trauma Amputation/Limb Salvage (METALS) study reported that participants with amputation not only had better functional outcomes but were more likely to engage in vigorous sports and a lesser probability of developing post-traumatic stress disorder [24]. However, the treatment priorities of soldiers may not be representative of the general population. 

The studies reveal mixed results on whether the functional or psychological outcome is convincingly superior for limb salvage compared to amputation. Nevertheless, there is a trend towards improved functional outcomes with amputation whereas psychological outcomes are better with limb salvage. This discrepancy suggests that there is a heavy involvement of patient factors, such as their demeanour and treatment goals, in determining the overall success of the management option. In patients for whom functional recovery is a priority and would prefer to have a shorter rehabilitation period and earlier return to work, an option to amputate may be preferable. Conversely, cultural and religious priorities vary across the world and in some developing countries, amputation is often not considered an option [1].

## Conclusions

The decision to either amputate or attempt limb salvage remains a conundrum. Although the various scoring systems can be used as supplementary tools, they shouldn’t be solely relied upon in this difficult decision-making process. Their advantages are constrained since they rely on retrospective data from small patient populations and, hence, a number of other factors should be taken into account that includes both patient and clinical outcomes. This clinical judgement should also take into consideration future functionality, available recovery programs, the patient’s demeanour, and also the surgeon’s enthusiasm and skill. Given the advances in technology, technical viability does not appear to be a sufficient basis for salvage and one must question whether the final result of the limb salvage procedure and its associated ongoing complications will be functionally acceptable to the patient and lead to their best outcome.
